# Tenofovir vs Entecavir Among Patients With HBV-Related HCC After Resection

**DOI:** 10.1001/jamanetworkopen.2023.40353

**Published:** 2023-10-31

**Authors:** Pengpeng Li, Yuanyuan Wang, Jian Yu, Judian Yu, Qifei Tao, Jinwei Zhang, Wan Yee Lau, Weiping Zhou, Gang Huang

**Affiliations:** 1 Third Affiliated Hospital of Naval Medical University, Eastern Hepatobiliary Surgery Hospital, Shanghai, China; 2 Faculty of Medicine, Chinese University of Hong Kong, Prince of Wales Hospital, Hong Kong Special Administrative Region, People’s Republic of China

## Abstract

**Importance:**

Tenofovir disoproxil and entecavir are both commonly used first-line antiviral treatments, but their comparative recurrence and overall survival (OS) benefits remain unclear.

**Objective:**

To explore differences of tenofovir disoproxil vs entecavir in recurrence-free survival (RFS) and OS after liver resection with curative intent in patients with hepatocellular cancer (HCC) related to hepatitis B virus (HBV).

**Design, Setting, and Participants:**

This retrospective cohort study was conducted at Eastern Hepatobiliary Surgery Hospital, a tertiary referral hospital in Shanghai, China, between January 4, 2015, and April 1, 2023. Participants included patients with HBV-related HCC who underwent liver resection with curative intent from January 2015 to December 2018. Patients who received tenofovir disoproxil were matched with patients who received entecavir in a 1:1 ratio using propensity score matching. Data were analyzed from April 3 to May 31, 2023.

**Exposures:**

Receiving tenofovir disoproxil or entecavir as antiviral treatment for HBV.

**Main Outcomes and Measures:**

Primary end points were RFS and OS rates.

**Results:**

Among 4451 patients (mean [SD] age, 58.1 [10.0] years; 3764 male [84.6%]; median [range] follow-up, of 51 [3 to 91] months), 989 patients in each of the groups were selected in propensity score matching. Baseline characteristics were comparable. In propensity score–matched groups, OS rates were 92.2% at 1 year, 70.9% at 3 years, and 54.2% at 5 years in the entecavir group, compared with 90.9% at 1 year, 75.2% at 3 years, and 64.0% at 5 years in the tenofovir disoproxil group. RFS rates were 83.9% at 1 year, 50.0% at 3 years, and 43.3% at 5 years in the entecavir group, compared with 85.3% at 1 year, 55.6% at 3 years, and 51.4% at 5 years in the tenofovir disoproxil group. Patients in the tenofovir disoproxil group had better OS (hazard ratio, 0.82; 95% CI, 0.72 to 0.94; *P* = .004) and RFS rates (hazard ratio, 0.81; 95% CI, 0.72 to 0.92; *P* = .001) compared with the entecavir group. Restricted mean survival time differences of entecavir vs tenofovir disoproxil groups were −0.05 (95% CI, −0.18 to 0.08) months at 1 year (*P* = .45), 0.20 (95% CI, −0.62 to 1.03) months at 3 years (*P* = .63), and 1.82 (95% CI, 0.14 to 3.51) months at 5 years (*P* = .03).

**Conclusions and Relevance:**

These findings suggest that in patients undergoing curative liver resection for HBV-related HCC, tenofovir disoproxil was associated with better long-term OS and RFS rates compared with entecavir, providing insights for antiviral treatment.

## Key Points

**Question:**

What are the recurrence-free and overall survival rates associated with tenofovir disoproxil vs entecavir among patients with hepatocellular cancer (HCC) related to hepatitis B virus (HBV) after liver resection with curative intent?

**Findings:**

In this cohort study of 4451 patients with HBV-related HCC who underwent hepatectomy, tenofovir disoproxil treatment was associated with significantly better long-term overall survival and recurrence-free survival rates compared with entecavir treatment.

**Meaning:**

These findings suggest that for postoperative patients with HBV-related HCC, tenofovir disoproxil could be considered the preferred antiviral treatment.

## Teaser

This cohort study explores differences in recurrence-free survival and overall survival associated with tenofovir disoproxil vs entecavir after liver resection with curative intent in patients with hepatocellular cancer (HCC) related to hepatitis B virus (HBV).

## Introduction

Hepatocellular carcinoma (HCC) is one of the most common cancers worldwide, and viral hepatitis remains an important etiological factor.^1^ Surgical resection is still the most commonly used first-line treatment aiming to cure patients with resectable tumors and preserved liver function.^2^ However, a significant proportion of patients develop tumor recurrence after liver resection, leading eventually to death. HCC related to hepatitis B virus (HBV) is common in Asia and Africa, and the disease can be associated with high HBV viral loads.^3^ Antivirus therapy has been shown to prevent tumor recurrence and prolong overall survival (OS) after liver resection, and it is now the cornerstone in treating liver cancer in patients with both high and low HBV DNA levels.^4,5^ Both tenofovir disoproxil and entecavir are widely used as the first-line antiviral treatment for patients with HBV-related HCC.^6^ It remains controversial whether these drugs result in different clinical benefits in terms of recurrence-free survival (RFS) and OS after liver resection.^7,8,9,10^ Thus, this study was conducted to assess the association of tenofovir disoproxil vs entecavir with RFS and OS after liver resection with curative intent in a large cohort of patients with HBV-related HCC.

## Methods

### Study Design

This cohort study was approved by the institutional review board of the Third Affiliated Hospital of Naval Medical University, Shanghai, China, with an exemption for informed consent because exposures were within routine clinical practice. The study followed the Strengthening the Reporting of Observational Studies in Epidemiology (STROBE) reporting guideline for observational studies. This retrospective study was conducted among 5336 consecutive patients with HBV-related HCC who underwent liver resection with curative intent at Eastern Hepatobiliary Surgery Hospital, a tertiary referral hospital, from January 2015 to December 2018. The follow-up outcome ascertainment was completed on April 1, 2023.

### Patients

The inclusion criteria were patients aged 18 to 80 years with HBV-related histopathologically confirmed HCC, who underwent radical liver resection (defined as no tumor remaining in the liver or any other sites of the body and having negative histopathological resection margins), started entecavir or tenofovir disoproxil as antiviral therapy within 3 months before the diagnosis of HBV-related HCC or within 1 month after the operation, and continued antiviral treatment after the operation. The exclusion criteria were patients who had other histopathological tumors other than HCC, a history of other antiviral therapy apart from tenofovir disoproxil or entecavir, other combined types of hepatitis virus infection, portal vein or hepatic vein tumor thrombosis or extrahepatic metastases, follow-up of less than 3 months, initiated antiviral treatments earlier than 3 months before the diagnosis of HBV-related HCC or later than 1 month after the operation, and received resection and tumor ablation simultaneously.

### Data Collection and Follow-Up

Routine patients’ baseline characteristics including sex, age, HBV infection status, antivirus regimen, α-fetoprotein (AFP) level, platelet count, liver function tests, complete blood count, coagulation profile, maximum tumor diameter, tumor number (single or multiple), cirrhosis status, presence of tumor encapsulation, surgical resection margins, and microvascular invasion (MVI) were retrospectively collected. Intraoperative data collected in this study included operative procedures, intraoperative blood loss, and volume of blood transfusion.

Patients were regularly followed-up after the operation in the HCC clinic once every 3 months in the first year, and half-yearly thereafter. Patients who failed to attend the scheduled follow-up appointments were contacted by a research nurse. Patients in each group adhered to their original antiviral regimens (entecavir or tenofovir disoproxil) unless a viral breakthrough was found during follow-up. The drug switch followed the instructions stated in the American Association for the Study of Liver Diseases guidelines for treatment of chronic hepatitis B.^11^

The degrees of liver fibrosis were evaluated using METAVIR scoring.^12^ Surgical resection margin was defined as the shortest macroscopic distance from the edge of tumor to the resection plane. R0 resection was defined as no gross tumor left after resection with nonexistence of tumor cells at the liver transection plane on microscopic examination. The primary end points were the RFS and OS rates after liver resection with curative intent. OS was defined as the time from first treatment to death of all causes. RFS was defined as the period during which a patient remained free from tumor recurrence after liver resection with curative intent.

### Statistical Analysis

Multiple imputation by chained equations was used to impute the missing data, and the imputation number was increased to 25. Details on the missing data before and after the imputation are provided in eTable 1 in Supplement 1. Multiple data sets were merged by Rubin rules. To reduce selection bias arising from lack of randomization, propensity score matching was performed to match patients who received tenofovir disoproxil with patients who received entecavir in a 1:1 ratio using the greedy nearest-neighbor matching strategy, with a caliper of 0.2 times the pooled estimate of the SD of the propensity score based on a priori selected baseline characteristics of age, sex, positivity for HBV e-antigen, HBV DNA level, AFP level, platelet count, total bilirubin level, alanine transaminase (ALT) level, albumin (ALB) level, prothrombin time (PT), maximum tumor diameter, tumor number (single or multiple), cirrhosis status, presence of tumor encapsulation, surgical resection margins, blood loss, and MVI. Covariate balance was assessed by the standardized mean difference and was considered good when the absolute standardized mean difference was less than 0.1. Additional sensitivity analyses were conducted to assess stability of the model by using the complete unmatched cohort without missing data, unmatched cohort without matching, 1:3 nearest-neighbor matching cohort, and covariate adjustment of Cox regression with propensity score as the only covariate. Continuous data were expressed as median (range), and categorical data were expressed as a count or ratio. The Mann-Whitney *U* test was used as the nonparametric test for 2 independent samples. Categorical variables were tested by the Pearson χ^2^ or Fisher exact tests. The Kaplan-Meier method was used to estimate OS. Log-rank test was used to compare survival outcomes between groups. Cox regressions were carried out to examine the associations between OS and demographic and other covariates. Restricted mean survival time (RMST) was used to estimate survival benefits. All computations relied on the SAS version 9.4 (SAS Institute) and R version 4.1.3 (R Project for Statistical Computing). A 2-sided *P* < .05 was considered statistically significant. Data were analyzed from [placeholder] to [placeholder].

## Results

**Figure 1.**
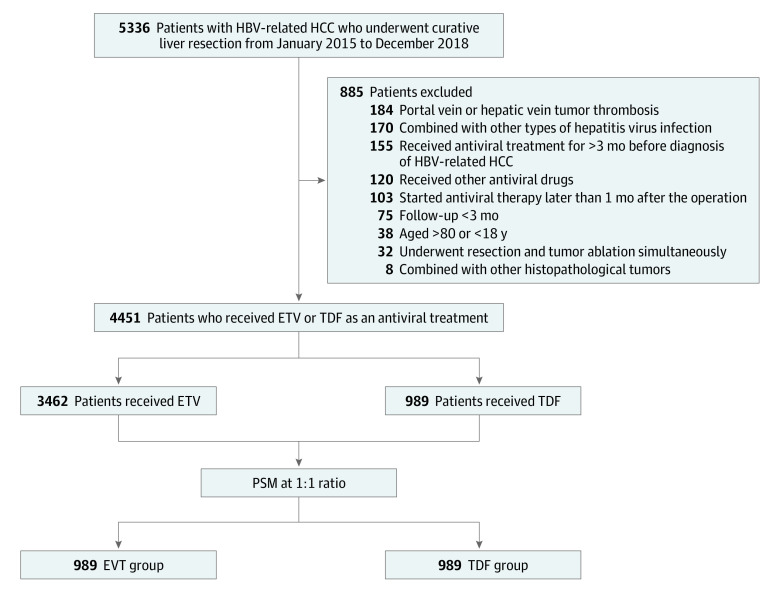
Flowchart of Patient Selection ETV indicates entecavir; HBV, hepatitis B virus; HCC, hepatocellular cancer; and TDF, tenofovir disoproxil.

From January 2015 to December 2018, a total of 4451 patients (mean [SD] age, 58.1 [10.0] years; 3764 [84.6%] male) with HBV-related HCC underwent liver resection with curative intent in our institution, including 3462 patients receiving entecavir and 989 patients receiving tenofovir disoproxil, and were considered eligible for propensity score matching at a 1:1 ratio. After PSM, 989 pairs of patients were matched and were included into each of the 2 groups (Figure 1). A total of 52 patients in the entecavir group experienced viral breakthrough and switched to tenofovir disoproxil. No viral breakthrough was observed in patients who used tenofovir disoproxil as antiviral treatment during follow-up.

### Baseline Characteristics

The baseline characteristics of the patients before and after matching are shown in Table 1. After matching, the absolute standardized mean differences of all variables were less than 0.1, indicating a good balance in baseline characteristics between groups.

**Table 1.** Baseline Characteristics of Patients Before and After Propensity Score Matching

Characteristics	Unmatched cohort				Propensity score–matched cohort			
	Patients, No. (%)		SMD	*P* value	Patients, No. (%)		SMD	*P* value
	Entecavir (n = 3462)	TDF (n = 989)			Entecavir (n = 989)	TDF (n = 989)		
Age, mean (SD), y	58.1 (9.9)	58.4 (10.5)	0.030	.40	58.3 (9.8)	58.4 (10.5)	0.004	.93
Sex								
Male	2913 (84.1)	851 (86.0)	NA	.14	844 (85.3)	851 (86.0)	NA	.65
Female	549 (15.9)	138 (14.0)	NA		145 (14.7)	138 (14.0)	NA	
AFP, mean (SD), ng/mL	500.3 (4723.1)	461.1 (3065.8)	−0.003	.93	543.2 (6339.4)	461.1 (3065.8)	0.032	.48
PT, mean (SD), s	11.8 (0.9)	11.8 (0.9)	−0.083	.02	11.8 (0.8)	11.8 (0.9)	−0.019	.67
Positive for HBV e antigens	890 (25.7)	261 (26.4)	−0.016	.67	270 (27.3)	261 (26.4)	0.021	.65
HBV DNA, mean (SD), log copies/mL^a^	3.3 (1.8)	3.3 (1.8)	0.009	.80	3.3 (1.8)	3.3 (1.8)	0.012	.79
ALT, mean (SD), U/L	29.9 (12.9)	29.1 (12.8)	−0.059	.11	29.2 (12.8)	29.1 (12.8)	−0.006	.89
ALB, mean (SD), g/dL	4.3 (0.5)	4.3 (0.5)	0.017	.64	4.3 (0.5)	4.3 (0.5)	0.017	.70
Total bilirubin, mean (SD), mg/dL	0.9 (0.3)	0.9 (0.3)	−0.037	.30	0.9 (0.3)	0.9 (0.3)	0.010	.83
Platelet count, mean (SD), 10^9^/L	158.3 (73.0)	159.5 (72.3)	0.017	.65	160.6 (73.3)	159.5 (72.3)	−0.015	.74
Liver cirrhosis	1352 (39.1)	362 (36.6)	0.051	.16	360 (36.4)	362 (36.6)	−0.004	.93
Maximum tumor size, median (range), cm	4.2 (0.3-27.8)	4.1 (0.5-23.1)	0.042	.23	4.2 (0.3-25.0)	4.1 (0.5-23.1)	<-0.001	.99
Multiple tumors	535 (15.5)	166 (16.8)	−0.036	.31	178 (18.0)	166 (16.8)	0.033	.48
Tumor capsule	2182 (63.0)	596 (60.3)	0.057	.11	588 (59.5)	596 (60.3)	−0.017	.71
MVI grade								
0	1954 (56.4)	518 (52.4)	NA	.02	512 (51.8)	518 (52.4)	NA	.82
1	887 (25.6)	271 (27.4)	−0.040		276 (27.9)	271 (27.4)	0.011	
2	621 (17.9)	200 (20.2)	−0.058		201 (20.3)	200 (20.2)	0.003	
BCLC stage								
0	362 (10.5)	120 (12.1)	NA	.25	98 (9.9)	120 (12.1)	NA	.18
A	2680 (77.4)	743 (75.1)	NA		747 (75.5)	743 (75.1)	NA	
B	420 (12.1)	126 (12.7)	NA		144 (14.6)	126 (12.7)	NA	
Resection margin, mean (SD), mm	0.7 (0.7)	0.7 (0.7)	−0.026	.47	0.7 (0.6)	0.7 (0.7)	−0.002	.97
Blood loss, mean (SD), log mL^a^	5.4 (0.7)	5.4 (0.7)	0.027	.46	5.4 (0.7)	5.4 (0.7)	0.008	.86
Blood transfusion, median (range), mL	0 (0-8600)	0 (0-5200)	NA	.76	0 (0-4410)	0 (0-5200)	NA	.45
Follow-up, median (range), mo	51 (3-91)	51 (3-91)	NA	NA	50 (3-91)	50 (3-91)	NA	NA

Abbreviations: AFP, α-fetoprotein; ALB, albumin; ALT, alanine aminotransferase; BCLC, Barcelona Clinic Liver Cancer; HBV, hepatitis B virus; MVI, microvascular invasion; PT, prothrombin time; SMD, standardized mean difference; TDF, tenofovir disoproxil.

SI conversion factors: To convert AFP to micrograms per liter, multiply by 1; ALB to grams per liter, multiply by 10; ALT to microkatals per liter, multiply by 0.0167; total bilirubin to micromoles per liter, multiply by 17.104.

^a^ Values were log-transformed with a base of 10.

### Postoperative Tumor RFS and OS 

In the unmatched cohort of 4451 patients, 3423 patients (76.9%) completed the 5-year follow-up. The OS rates were 91.7% at 1 year, 73.4% at 3 years, and 59.2% at 5 years, and RFS rates were 85.4% at 1 year, 54.1% at 3 years, 47.9% at 5 years. When comparing groups, OS rates in the entecavir group were 91.9% at 1 year, 72.9% at 3 years, and 57.8% at 5 years, vs 90.9% at 1 year, 75.2% at 3 years, and 64.0% at 5 years in the tenofovir disoproxil group. RFS rates in the entecavir group were 85.4% at 1 year, 53.6% at 3 years, and 47.0% at 5 years, vs 85.3% at 1 year, 55.6% at 3 years, and 51.4% at 5 years in the tenofovir disoproxil group. Hazard ratios (HRs) indicated no difference in OS (HR, 0.91 [95% CI, 0.81 to 1.01]; *P* = .09) and significantly better RFS (HR, 0.90 [95% CI, 0.81 to 1.00]; *P* = .04) in the tenofovir disoproxil group vs the entecavir group. Due to violation of the proportional hazards assumption, RMST was used for interpretation. The RMST (SD) for OS was 11.65 (0.02) months at 1 year, 31.30 (0.15) months at 3 years, and 47.03 (0.32) months at 5 years in the entecavir group and 11.59 (0.05) months at 1 year, 31.17 (0.30) months at 3 years, and 47.73(0.61) months at 5 years in the tenofovir disoproxil group.

**Figure 2.**
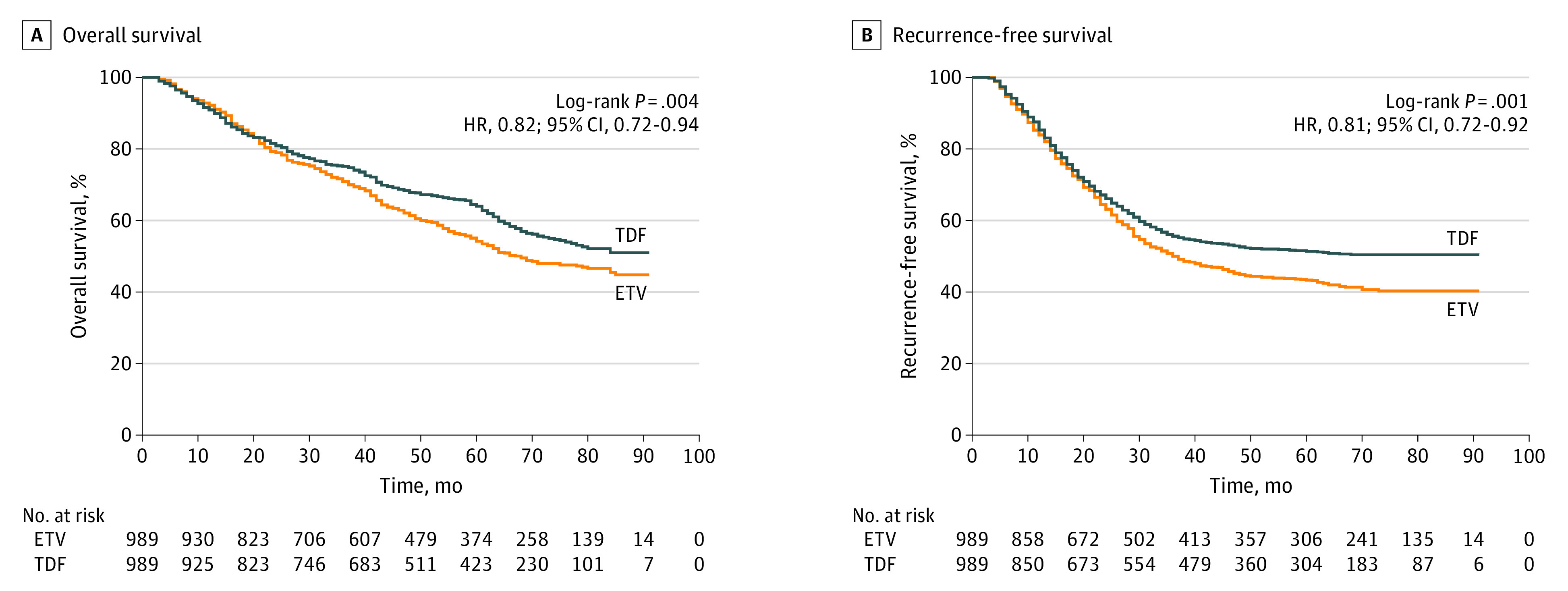
Overall Survival and Recurrence-Free Survival in the Propensity Score–Matched Cohort ETV indicates entecavir; HR, hazard ratio; and TDF, tenofovir disoproxil.

In the propensity score–matched cohort with a median (range) follow-up of 50 (3 to 91) months, the overall OS rates for 1978 patients were 91.6% at 1 year, 73.0% at 3 years, and 59.1% at 5 years, and the RFS rates were 84.6% at 1 year, 52.8% at 3 years, and 47.3% at 5 years. Among patients who received entecavir , OS rates were 92.2% at 1 year, 70.9% at 3 years, and 54.2% at 5 years, compared with 90.9% at 1 year, 75.2% at 3 years, and 64.0% at 5 years in patients who received tenofovir disoproxil; RFS rates were 83.9% at 1 year, 50.0% at 3 years, and 43.3% at 5 years among patients who received entecavir, compared with 85.3% at 1 year, 55.6% at 3 years, and 51.4% at 5 years in the tenofovir disoproxil group (Figure 2). Hazard ratios showed significantly better OS (HR, 0.82 [95% CI, 0.72-0.94]; *P* = .004) and RFS (HR, 0.81 [95% CI, 0.72 to 0.92]; *P* = .001) in the tenofovir disoproxil group compared with the entecavir group.

**Figure 3.**
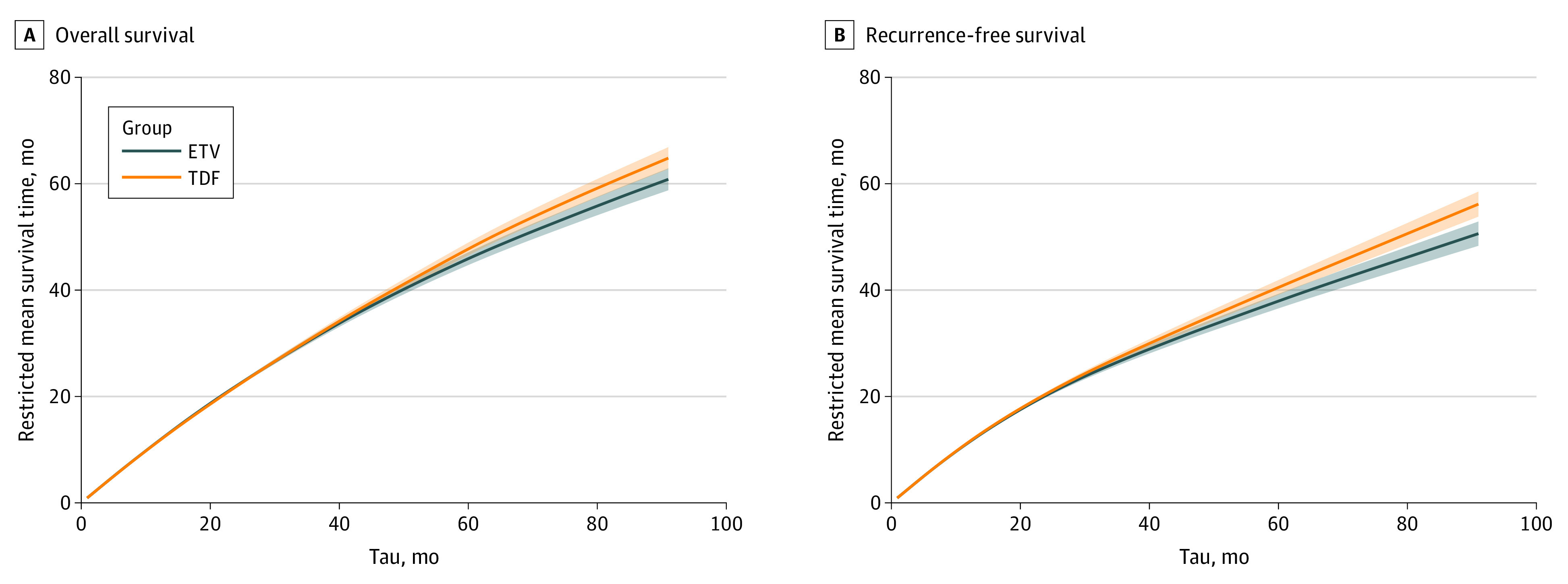
Restricted Mean Survival Times (RMST) in the Propensity Score–Matched Cohort ETV indicates entecavir; TDF, tenofovir disoproxil.

The RMST (SD) for OS was 11.64 (0.05) months at 1 year, 30.97 (0.29) months at 3 years, and 45.91 (0.61) months at 5 years in the entecavir group, compared with 11.59 (0.05) months at 1 year, 31.17 (0.30) months at 1 year, and 47.73 (0.61) months at 5 years in the tenofovir disoproxil group (Figure 3). The RMST differences for OS of the entecavir group vs the tenofovir disoproxil group were not significantly different at 1 year (−0.05 [95% CI, −0.18 to 0.08] months; *P* = .45) or 3 years (0.20 [95% CI, −0.62 to 1.03] months; *P* = .63). However, a significant difference was observed at 60 months after liver resection (1.82 [95% CI, 0.14 to 3.51] months; *P* = .03).

The linear model for RMST differences showed factors associated with better OS at 1 year included younger age, lower AFP level, lower PT, solitary tumor, presence of tumor encapsulation, smaller tumor, and better than grade 2 MVI (eTable 2 in Supplement 1). At 3 years, the linear model for RMST differences found that factors associated with better OS included younger age, lower AFP level, higher platelet counts, solitary tumor, tumor encapsulation, smaller tumor, better than grade 1 MVI, and better than grade 2 MVI (eTable 2 in Supplement 1). At 5 years, the linear model for RMST differences found that factors associated with better OS included younger age, lower AFP level, higher platelet counts, lower total bilirubin level, no cirrhosis, solitary tumor, tumor encapsulation, smaller tumor, better than grade 1 MVI, better than grade 2, and using tenofovir disoproxil (eTable 2 in Supplement 1).

The RMST (SD) for RFS was 11.36 (0.06) months at 1 year, 26.94 (0.36) months at 3 years, and 37.92 (0.70) months at 5 years in the entecavir group and 11.45 (0.05) months at 1 year, 27.76 (0.35) months at 3 years, and 40.49 (0.71) months at 5 years in the tenofovir disoproxil group (Figure 3). On follow-up, the RMST differences for RFS of the entecavir group vs the tenofovir disoproxil group were not significantly different until 5 years after surgery (2.57 [95% CI, 0.62-4.51] months; *P* = .01), suggesting that the tenofovir disoproxil group had better late RFS rates compared with the entecavir group.

Multiple additional sensitivity analyses, including unmatched cohort, unmatched cohort without missing data, 1:3 nearest-neighbor matching, and covariate adjustment of Cox regression with propensity score as the only covariate in the 2 adjustment models yielded similar results (Table 2). Exploratory subgroup analyses based on the Barcelona Clinic Liver Cancer stage system indicated that a statistically significant difference in the RMST between the entecavir and tenofovir disoproxil groups at 5 years was only observed within the stage B subgroup (eTable 3 in Supplement 1).

**Table 2.** Multiple Additional Sensitivity Analyses in Different Cohorts

**OS model**	**RMST difference (95% CI), mo**	***P* value**
**Unmatched cohort^a^**		
Without covariate adjustment		
12 mo	−0.06 (−0.16 to 0.05)	.29
36 mo	−0.13 (−0.79 to 0.53)	.70
60 mo	0.71 (−0.64 to 2.06)	.30
Adjusted for age and sex		
12 mo	−0.06 (−0.16 to 0.05)	.28
36 mo	−0.13 (−0.79 to 0.54)	.71
60 mo	0.71 (−0.65 to 2.06)	.31
Adjusted for all covariates^b^		
12 mo	−0.02 (−0.12 to 0.07)	.63
36 mo	0.24 (−0.30 to 0.78)	.39
60 mo	1.55 (0.48 to 2.61)	.005
Adjusted for propensity score only		
12 mo	−0.02 (−0.13 to 0.08)	.66
36 mo	0.25 (−0.40 to 0.89)	.45
60 mo	1.57 (0.26 to 2.87)	.02
**Unmatched cohort without missing data** ^c^		
Without covariate adjustment		
12 mo	−0.03 (−0.14 to 0.08)	.58
36 mo	0.15 (−0.53 to 0.83)	.66
60 mo	1.44 (0.05 to 2.82)	.04
Adjusted for age and sex		
12 mo	−0.03 (−0.14 to 0.08)	.57
36 mo	0.16 (−0.53 to 0.84)	.65
60 mo	1.44 (0.05 to 2.83)	.04
Adjusted for all covariates^b^		
12 mo	−0.01 (−0.11 to 0.09)	.91
36 mo	0.43 (−0.14 to 1.00)	.14
60 mo	1.70 (0.54 to 2.85)	.004
**PSM cohort at a ratio of 1:3** ^d^		
Without covariate adjustment		
12 mo	−0.04 (−0.15 to 0.07)	.47
36 mo	0.04 (−0.64 to 0.72)	.91
60 mo	1.22 (−0.17 to 2.60)	.09
Adjusted for age and sex		
12 mo	−0.04 (−0.15 to 0.07)	.46
36 mo	0.04 (−0.64 to 0.72)	.91
60 mo	1.21 (−0.17 to 2.60)	.09
Adjusted for all covariates		
12 mo	−0.02 (−0.12 to 0.08)	.64
36 mo	0.22 (−0.33 to 0.78)	.43
60 mo	1.64 (0.55 to 2.73)	.003

Abbreviations: OS, overall survival; PSM, propensity score matching; RMST, restricted mean survival time.

^a^ Includes 3462 patients in the entecavir group and 989 patients in the tenofovir disoproxil group.

^b^ Included age, sex, positivity for hepatitis B e-antigen, hepatitis B virus DNA level, α-fetoprotein level, platelet count, total bilirubin level, alanine transaminase level, albumin level, prothrombin time, maximum tumor diameter, tumor number (single or multiple), cirrhosis status, presence of tumor encapsulation, surgical resection margins, blood loss, microvascular invasion, and year of surgery.

^c^ Includes 3185 patients in the entecavir group and 909 patients in the tenofovir disoproxil group.

^d^ Includes 2848 patients in the entecavir group and 989 patients in the tenofovir disoproxil group.

## Discussion

This cohort study aimed to investigate any potential differences in long-term survival in patients with HBV-related HCC after resections with curative intent by use of either entecavir or tenofovir disoproxil, which are regarded as the mainstream antiviral medications in clinical practice. The basic research data, derived from a large cohort of 5336 patients with HBV-related HCC patients who underwent liver resections with curative intent in a tertiary hepatic surgery center, were well balanced by using propensity score matching to minimize selection biases. After PSM, there were 989 patients in each of the 2 groups. Due to the intertwined survival curves at the beginning of follow-up, RMST was used to evaluate survival benefits in different time frames. The study found that tenofovir disoproxil was associated with lower RFS and better OS rates in on long-term follow-up before and after propensity score matching.

In the propensity score–matched cohorts, the RFS curves for the entecavir and tenofovir disoproxil groups intertwined in the early follow-up period up to 20 months, and then they gradually separated, when a significant difference between groups became evident at 5 years. A similar trend was also observed in the OS curves. A possible explanation can be based on a widely accepted theory that most early HCC recurrences occurring within 2 years of hepatectomy come from the original tumors metastasizing to the remnant liver.^13^ The similarity in intrahepatic tumor transmission, mainly from the similar tumor-related characteristics in the 2 group of patients, contributed to the tangled survival curves on short-term follow-up. On the other hand, differences in new tumor genesis in the remnant liver due to differences in liver inflammatory status and fibrotic changes associated with different antiviral agents were likely responsible for the late separation of survival curves on long-term follow-up.

Although the potential mechanisms on why there were significantly better RFS and OS rates in the tenofovir disoproxil group compared with the entecavir group have not been clarified, a 2018 study by Murata et al^14^ found that a nucleotide analogue (tenofovir disoproxil) induced a higher expression of interferon-λ3 and inhibited production of HBV antigen, which was not seen in the nucleoside analogue (entecavir).^14^ Studies have found that interferon-λ3 exhibits antitumor activity in murine models of cancer^15,16^ and also inhibits tumor growth directly by inducing apoptosis and cell cycle arrest to enhance host immunity.^17^ These studies can in part explain the difference in OS and RFS between groups in this study. Furthermore, entecavir has been shown to be capable of damaging DNA, which is known to have a genotoxic effect^18^ and can be a presumed mechanism for carcinogenesis.

The prognosis of patients with HCC, thanks to early diagnosis and new therapeutic approaches, has been improving gradually over time.^19^ However, long-term survival is still low due to the high HCC recurrence rate after liver resection. Recurrence occurs in approximately 70% of patients by 5 years after liver resection with curative intent.^3^ These recurrences include early recurrence within 2 years of hepatectomy and late recurrence after that time.^13^ There are many factors, including tumor size, AFP level before hepatectomy, presence of MVI, degree of liver fibrosis, HBV DNA replication level, and others, that have been shown to contribute to intrahepatic recurrence.^3,20,21^ Of these inherent factors, HBV DNA replication level is a factor that can be lowered by antiviral therapy. Currently, there is a consensus that antiviral treatment should be given to all patients with HBV-related HCC preoperatively and/or postoperatively to reduce postoperative recurrence and to improve OS, regardless of the HBV DNA level.^4,5,22,23^ Entecavir and tenofovir disoproxil are potent agents with high genetic barriers for the virus to develop drug resistance. These drugs have sustained HBV suppression, leading to a lower risk of HCC development and recurrence compared with other drugs with low barriers against HBV resistance (eg, adefovir dipivoxil, lamivudine, telbivudine).^11,24,25^ Apart from HBV suppression, tenofovir disoproxil also exhibits antitumor properties.^26,27^ However, no guidelines for antiviral treatment in patients with chronic HBV or HBV-related HCC state any preference for entecavir or tenofovir disoproxil. Previous studies have had mixed results regarding the risks of HCC recurrence between using entecavir and tenofovir disoproxil, with superior RFS rates associated with tenofovir disoproxil reported in some studies,^7,8,10,28,29,30^ which was consistent with our findings, but not in other studies.^9,31,32^ However, to our knowledge, no study has shown the superiority of entecavir over tenofovir disoproxil. Among 3 studies^9,31,32^ that showed no significant difference in RFS, 2 included patients who underwent radiofrequency ablation as an anti-HCC treatment.^31,32^ Early intrahepatic HCC recurrence rates after radiofrequency ablation have been reported to be significantly higher than recurrence rates after liver resection due to inadequately ablated tumors, thus masking the differences in efficacy of antirecurrence between entecavir and tenofovir disoproxil. The similarity in RFS between entecavir and tenofovir disoproxil presented in the study by Wang et al^9^ could be explained by the fact that the authors excluded patients with incomplete clinical data other than imputing missing data by using statistical approaches, and this can lead to selection bias.

### Limitations

This study has some limitations. The major limitation of this study is that it was a retrospective cohort study, which has the inherent defects of introducing selection bias. To minimize any potential bias, propensity score matching, sensitivity analyses, multivariable adjustment, and RMST were used. However, not all confounding factors that were associated with HCC recurrence could be completely adjusted for using the statistical models included in this analysis. Second, all the patients in this study came from a single institution in Shanghai, China, where HBV with the genotype B and C were predominant. Whether the outcomes in this study can be generalized to other populations with other prevailing HBV genotypes for HCC remain unclear. Third, there were some disparities in the onset time of antiviral therapy, although in this study the initiation of antiviral therapy was restricted to before or within 1 month after patients underwent operations. Drug switching between agents existed in our study; therefore, the intention to treat criteria was used to interpret the outcomes.

## Conclusions

This cohort study found that among patients with HBV-related HCC who underwent liver resections with curative intent, tenofovir disoproxil was associated with significantly better OS and RFS rates compared with entecavir on long-term follow-up but not in short-term follow-up. Tenofovir disoproxil could be considered the preferred long-term antiviral treatment for these patients.

## Notes

Supplement 1. **eTable 1.** Distributions of Variables With Missing Data Comparing Observed Complete Case Data to Results From Pooling the Datasets With Imputed Variables From Multiple Imputation **eTable 2.** Linear Model Analyses on RMST Differences for Better Overall Survival Rates at Different Times **eTable 3.** Subgroup Analyses Based on BCLC Stage System

Supplement 2.
Data Sharing Statement
