# Supplementary material for: Tenofovir vs Entecavir Among Patients With HBV-Related HCC After Resection
Source: JAMA Netw Open. 2023 Oct 31;6(10):e2340353. doi: 10.1001/jamanetworkopen.2023.40353 (PMC10618847; doi:10.1001/jamanetworkopen.2023.40353)
Supplement: Supplement 1. — eTable 1. Distributions of Variables With Missing Data Comparing Observed Complete Case Data to Results From Pooling the Datasets With Imputed Variables From Multiple Imputation eTable 2. Linear Model Analyses on RMST Differences for Better Overall Survival Rates at Different Times eTable 3. Subgroup Analyses Based on BCLC Stage System [file jamanetwopen-e2340353-s001.pdf]

Supplemental Online Content

Li P, Wang Y, Yu J, et al. Tenofovir vs entecavir among patients with HBV-related HCC after resection. *JAMA Netw Open*. 2023;6(10):e2340353. doi:10.1001/jamanetworkopen.2023.40353

eTable 1. Distributions of Variables With Missing Data Comparing Observed Complete Case Data to Results From Pooling the Datasets With Imputed Variables From Multiple Imputation
eTable 2. Linear Model Analyses on RMST Differences for Better Overall Survival Rates at Different Times
eTable 3. Subgroup Analyses Based on BCLC Stage System

This supplemental material has been provided by the authors to give readers additional information about their work.

© 2023 Li P et al. *JAMA Network Open.*

eTable 1. Distributions of variables with missing data comparing observed complete case data to results from pooling the datasets with imputed variables from multiple imputation

Characteristics	Number with missing data, n (%)	Complete case, median (ranges)	Multiple imputation, median (ranges)
**AFP (Log mg/mL)[a]**	12 (0.3)	3.5 (-0.5-12.2)	3.5 (-0.5-12.2)
**PT (Seconds)**	51 (1.1)	11.7 (9.1-14.1)	11.7 (9.1-14.1)
**HBV-DNA (Log copies/ml)[a]**	66 (1.5)	2.5 (1.7-8.6)	2.5 (1.7-8.6)
**ALT (U/L)**	78 (1.8)	28.0 (4.0-60.0)	28.0 (4.0-60.0)
**ALB (g/L)**	78 (1.8)	42.5 (27.9-69.8)	42.5 (27.0-69.8)
**TBIL (umol/L)**	78 (1.8)	13.8 (3.0-35.7)	13.8 (3.0-35.7)
**Platelet count, (10^9/L)**	65 (1.5)	148.5 (16.0-555.0)	149.0 (15.0-555.0)
**Tumor Capsule, n (%)**	40 (0.9)	2552 (62.3%)	2778 (61.0%)
**Resection margin (mm)**	45 (1.0)	0.5 (0.0-9.0)	0.5 (0.0-9.0)

[a] Values were log-transformed with a base of 10.

AFP: Alpha-Fetoprotein

PT: Prothrombin time

HBe Ag: Hepatitis B e antigen

HBV-DNA: Hepatitis B virus Deoxyribonucleic acid

© 2023 Li P et al. *JAMA Network Open.*

TBIL: Total Bilirubin

ALT: Alanine aminotransferase

ALB: Albumin

eTable 2. Linear model analyses on RMST differences for better overall survival rates at different times

Characteristics	RMST differences (95% CI), months	P value
**One year**		
Age	-0.0044 (-0.0084 to - 0.0003)	P=.03
AFP level	-0.0353 (-0.0533 to - 0.0172)	P<.001
PT	-0.0531 (-0.1015 to - 0.0047)	P=.03
Solitary tumor	-0.3973 (-0.5490 to - 0.2456)	P<.001
Presence of tumor encapsulation	0.2069 (0.1217 to 0.2920)	P<.001
Tumor diameter	-0.0785 (-0.0970 to - 0.0599)	P<.001
Better than grade 2 MVI	-0.5652 (-0.7107 to - 0.4197)	P<.001
**Three years**		
Age	-0.0558 (-0.0792 to - 0.0324)	P<.001
AFP level	-0.2140 (-0.3215 to - 0.1065)	P<.001
Platelet counts	0.0064 (0.0028 to 0.0101)	P=.001
Solitary tumor	-2.5400 (-3.3219 to -1.7581)	P<.001
Presence of tumor encapsulation	1.4268 (0.9516 to 1.9021)	P<.001
Tumor diameter	-0.7563 (-0.8472 to - 0.6655)	P<.001
Better than grade 1 MVI	-1.8781 (-2.4339 to - 1.3223)	P<.001
Better than grade 2 MVI	-6.5501 (-7.3759 to - 5.7244)	P<.001
**Five years**		

© 2023 Li P et al. *JAMA Network Open.*

Age	-0.1080 (-0.1549 to - 0.0611)	P<.001
AFP level	-0.4556 (-0.6702 to - 0.2411)	P<.001
Platelet counts	0.0121 (0.0049 to 0.0192)	P=.001
Total bilirubin level	-0.0929 (-0.1740 to -0.0119)	P=.02
Cirrhosis	-1.9759 (-3.0378 to -0.9140)	P<.001
Solitary tumor	-4.8343 (-6.2857 to -3.3829)	P<.001
Presence of tumor encapsulation	1.9798 (1.0495 to 2.9102)	P<.001
Tumor diameter	-1.6381 (-1.8052 to -1.4711)	P<.001
Better than grade 1 MVI	-6.9655 (-8.1106 to -5.8204)	P<.001
Better than grade 2 MVI	-15.5009 (-17.0546 to -13.9471)	P<.001
Using tenofovir disoproxil	1.5631 (0.4937 to 2.6324)	P=.004

RMST: Restricted Mean Survival Time

AFP: α-fetoprotein

PT: prothrombin time

MVI: microvascular invasion

eTable 3. Subgroup analyses based on BCLC stage system

Characteristics	ETV group	TDF group	P value
**BCLC 0, n**	98	120	
RMST for OS (months)			
12	12.00	12.00	NA[a]
36	35.39	35.85	P=.17
60	55.99	58.29	P=.04

© 2023 Li P et al. *JAMA Network Open.*

RMST differences for OS (adjust for all covariates), differences (95% CI), months			
12	0		NA[a]
36	0.46 (-0.29- 1.20)		P=.23
60	1.81 (-0.32- 3.93)		P=.10
RMST for RFS (months)			
12	11.98	12	P=.31
36	32.36	33.78	P=.11
60	47.33	52.63	P=.02
RMST differences for RFS (adjust for all covariates), differences (95% CI), months			
12	0.03 (-0.02- 0.08)		P=.28
36	1.13 (-0.53- 2.79)		P=.18
60	4.45 (0.52- 8.37)		P=.03
**BCLC A, n**	747	743	
RMST for OS (months)			
12	11.73	11.68	P=.40
36	31.78	31.32	P=.33
60	47.44	47.97	P=.59
RMST differences for OS (adjust for all covariates), differences (95% CI), months			
12	-0.03 (-0.16- 0.10)		P=.64
36	-0.17 (-0.94- 0.61)		P=.67
60	1.18 (-0.37- 2.73)		P=.14
RMST for RFS (months)			
12	11.47	11.42	P=.55

© 2023 Li P et al. *JAMA Network Open.*

36	27.65	27.51	P=.80
60	39.31	39.92	P=.59
RMST differences for RFS (adjust for all covariates), differences (95% CI), months			
12	-0.01 (-0.16- 0.14)		P=.91
36	0.18 (-0.77- 1.13)		P=.71
60	1.17 (-0.78- 3.12)		P=.24
**BCLC B, n**	144	126	
RMST for OS (months)			
12	10.94	10.72	P=.46
36	23.74	25.80	P=.16
60	31.00	36.26	P=.05
RMST differences for OS (adjust for all covariates), differences (95% CI), months			
12	-0.24 (-0.76- 0.27)		P=.35
36	1.56 (-0.96- 4.08)		P=.22
60	4.78 (0.22- 9.35)		P=.04
RMST for RFS (months)			
12	10.34	11.07	P=.01
36	18.80	22.70	P=.01
60	22.86	30.63	P=.004
RMST differences for RFS (adjust for all covariates), differences (95% CI), months			
12	0.58 (0.03- 1.14)		P=.04
36	3.23 (0.46- 6.00)		P=.02
60	6.86 (1.81- 11.91)		P=.01

© 2023 Li P et al. *JAMA Network Open.*

NA[a]: Within the BCLC stage 0 subgroup, both the ETV and TDF groups had a one-year survival rate of 100%, with no observed mortality. As a result, no valid estimates could be derived.

ETV: Entecavir

TDF: Tenofovir disoproxil

RMST: Restricted Mean Survival Time

OS: Overall survival

RFS: Recurrence-free survival

Adjust for all covariates included: age, gender, positivity for hepatitis B e-antigen (HBeAg), HBV-DNA level, AFP level, platelet count, total bilirubin (TBIL) level, alanine transaminase (ALT) level, albumin (ALB) level, prothrombin time (PT), maximum tumor diameter, tumor number (single or multiple), cirrhosis status, presence of tumor encapsulation, surgical resection margins, blood loss, and microvascular invasion (MVI), and year of surgery.

© 2023 Li P et al. *JAMA Network Open.*
