# Supplementary material for: Tenofovir vs Entecavir Among Patients With HBV-Related HCC After Resection
Source: JAMA Netw Open. 2023 Oct 31;6(10):e2340353. doi: 10.1001/jamanetworkopen.2023.40353 (PMC10618847; doi:10.1001/jamanetworkopen.2023.40353)
Supplement: Supplement 2. — Data Sharing Statement [file jamanetwopen-e2340353-s002.pdf]

Data Sharing Statement

Li. Tenofovir vs Entecavir Among Patients With HBV-Related HCC After Resection. *JAMA Netw Open*. Published October 31, 2023. doi:10.1001/jamanetworkopen.2023.40353

Data
Data available: Yes
Data types: Deidentified participant data
How to access data: Please send an email to dragonlpp1984@163.com to request data
When available: With publication

Supporting Documents
Document types: None

Additional Information
Who can access the data: The data will be made available to all researchers who are interested in this topic.
Types of analyses: For research only.
Mechanisms of data availability: The data will be made available after approval of a proposal.
